# Cyclin G and the Polycomb Repressive complexes PRC1 and PR-DUB cooperate for developmental stability

**DOI:** 10.1371/journal.pgen.1007498

**Published:** 2018-07-11

**Authors:** Delphine Dardalhon-Cuménal, Jérôme Deraze, Camille A. Dupont, Valérie Ribeiro, Anne Coléno-Costes, Juliette Pouch, Stéphane Le Crom, Hélène Thomassin, Vincent Debat, Neel B. Randsholt, Frédérique Peronnet

**Affiliations:** 1 Sorbonne Université, Centre National de la Recherche Scientifique (CNRS), Institut de Biologie Paris-Seine (IBPS), Laboratory of Developmental Biology (LBD), Paris, France; 2 Institut de biologie de l’Ecole normale supérieure (IBENS), Ecole normale supérieure, CNRS, INSERM, PSL Université Paris Paris, France; 3 Sorbonne Université, Univ Antilles, Univ Nice Sophia Antipolis, CNRS, Evolution Paris Seine—Institut de Biologie Paris Seine (EPS - IBPS), Paris, France; 4 Institut de Systematique, Evolution, Biodiversité ISYEB UMR 7205, MNHN, CNRS, Sorbonne Université, EPHE, Muséum national d'Histoire naturelle, Sorbonne Universités, Paris, France; Centre National de la Recherche Scientifique, FRANCE

## Abstract

In *Drosophila*, ubiquitous expression of a short Cyclin G isoform generates extreme developmental noise estimated by fluctuating asymmetry (FA), providing a model to tackle developmental stability. This transcriptional cyclin interacts with chromatin regulators of the Enhancer of Trithorax and Polycomb (ETP) and Polycomb families. This led us to investigate the importance of these interactions in developmental stability. Deregulation of Cyclin G highlights an organ intrinsic control of developmental noise, linked to the ETP-interacting domain, and enhanced by mutations in genes encoding members of the Polycomb Repressive complexes PRC1 and PR-DUB. Deep-sequencing of wing imaginal discs deregulating *CycG* reveals that high developmental noise correlates with up-regulation of genes involved in translation and down-regulation of genes involved in energy production. Most Cyclin G direct transcriptional targets are also direct targets of PRC1 and RNAPolII in the developing wing. Altogether, our results suggest that Cyclin G, PRC1 and PR-DUB cooperate for developmental stability.

## Author summary

During development, the part of stochasticity inherent to biological processes induces noise. In animals with bilateral symmetry, developmental noise can be estimated by the variance in a population of the difference between the left and the right sides of individuals, the so-called fluctuating asymmetry (FA). The genetic bases of developmental stability, *i*.*e*. the control of developmental noise, are still unsolved. A large amount of data converges towards the importance of hubs–*i*.*e*. the most connected genes–in gene networks. Deregulating Cyclin G, a protein involved in transcription and in the cell cycle, induces high FA providing a unique system to address the genetic bases of developmental stability. Using this system, we show the existence of an organ intrinsic control of developmental noise linked to the interaction between Cyclin G and chromatin regulators. We also show that Cyclin G-induced FA correlates with the activation of genes involved in translation and the repression of genes involved in energy production. We suggest that fine-tuned control of these key functions is important for developmental stability

## Introduction

Developmental stability has been described as the set of processes that buffer disruption of developmental trajectories for a given genotype within a particular environment [1]. In other words, developmental stability compensates the random stochastic variation of processes at play during development. Many mechanisms working from the molecular to the whole organism levels contribute to developmental stability [2]. For example, chaperones, such as heat-shock proteins, participate in developmental stability by protecting misfolded proteins from denaturation in a large variety of processes [3–5]. In *Drosophila*, adjustment of cell growth to cell proliferation is essential to developmental stability by allowing to achieve a consistent organ size (*e*.*g*. wing size) in spite of variation in cell size or cell number [6,7].

Developmental noise, the “sum” of the stochastic part of each developmental process, can be observed macroscopically for morphological traits. In bilaterians, quantification of departure from perfect symmetry, the so-called fluctuating asymmetry (FA), is the most commonly used parameter to estimate developmental noise [8,9]. Indeed, the two sides of bilaterally symmetrical traits are influenced by the same genotype and environmental conditions, and differences between them are thus only due to developmental noise. The use of FA as an estimator of developmental noise makes analysis of the mechanistic and genetic bases of developmental stability compatible with custom genetic and molecular approaches of developmental biology.

The genetic bases of developmental stability remain unclear. Thus, its evolutionary role is subject to many speculations (for reviews see [7,10,11]). Experiments showing the role of *Hsp90* in buffering genetic variation led to the idea that developmental stability could be ensured by specific genes [12–15]. On the other hand, both theory and experiments show that complex genetic networks can become intrinsically robust to perturbations, notably through negative and positive feedbacks, suggesting that the topology of gene networks is of paramount importance for developmental stability [16]. Several authors have further suggested that hubs, *i*.*e*. the most connected genes in these networks, might be particularly important for developmental stability [17,18].

In *Drosophila*, mutants for *dILP8* and *Lgr3* involved in the control of systemic growth, have been reported to display high FA as compared to wild type flies, indicating that these genes are important for developmental stability [19–23]. Two studies have scanned the *Drosophila* genome for regions involved in developmental stability [24,25]. Several deletions increased FA but genes responsible for this effect inside the deletions were not identified. Nevertheless, these studies confirm that the determinism of developmental stability could be polygenic, as suggested by Quantitative Trait Loci analyses in mouse ([11] and references therein). Together, these data reinforce the idea that developmental stability depends on gene networks.

We have shown that the gene *Cyclin G* (*CycG*) of *D*. *melanogaster*, which encodes a protein involved in the cell cycle, is important for developmental stability [6,26,27]. Indeed, ubiquitous expression of a short Cyclin G version lacking the C-terminal PEST-rich domain (*CycG*^*ΔP*^) generates a very high FA in several organs, notably in the wing. Interestingly, FA induced by *CycG*^*ΔP*^ expression is associated with loss of correlation between cell size and cell number, suggesting that the noisy process would somehow be connected to cell cycle related cell growth [6]. Hence, *CycG* deregulation provides a convenient sensitized system to tackle the impact of cell growth on developmental stability.

*CycG* encodes a transcriptional cyclin and interacts with genes of the *Polycomb-group* (*PcG*), *trithorax-group* genes (*trxG*) and *Enhancer of Trithorax and Polycomb* (*ETP*) families [28]. These genes encode evolutionary conserved proteins assembled into large multimeric complexes that bind chromatin. They ensure maintenance of gene expression patterns during development (for a recent review see [29]). *PcG* genes are involved in long-term gene repression, whereas *trxG* genes maintain gene activation and counteract *PcG* action. *ETP* genes encode co-factors of both *trxG* and *PcG* genes, and behave alternatively as repressors or activators of target genes (for a review see [30]). More recently, we discovered that *CycG* behaves as an *Enhancer of Polycomb* regarding homeotic gene regulation suggesting that it is involved in the silencing of these genes [31]. Importantly, Cyclin G physically interacts with the ETP proteins Additional Sex Comb (Asx) and Corto *via* its N-terminal ETP-interacting domain, and co-localizes with them on polytene chromosomes at many sites. Hence, Cyclin G and these ETPs might share many transcriptional targets and might in particular control cell growth *via* epigenetic regulation of genes involved in growth pathways.

Here, we investigate in depth the role of *CycG* in developmental stability. We first show that localized expression of *CycG*^*ΔP*^ in wing imaginal discs is necessary to induce high FA of adult wings. Furthermore, this organ-autonomous effect increases when the ETP-interacting domain of Cyclin G is removed. We show that several mutations for *PcG* or *ETP* genes, notably those encoding members of the PRC1 and PR-DUB complexes, substantially increase *CycG*-induced FA. Next, we report analysis of the transcriptome of wing imaginal discs expressing *CycG*^*ΔP*^ by RNA-seq and find that transcriptional deregulation of genes involved in translation and energy production correlates with high FA of adult wings. By ChIP-seq, we identify Cyclin G binding sites on the whole genome in wing imaginal discs. Strikingly, we observe a significant overlap with genes also bound by Asx, by the Polycomb Repressive complex PRC1, and by RNAPolII in the same tissue. We identify a sub-network of 222 genes centred on Cyclin G showing simultaneous up-regulation of genes involved in translation and down-regulation of genes involved in mitochondrial activity and metabolism. Taken together, our data suggest that Cyclin G and the Polycomb complexes PRC1 and PR-DUB cooperate in sustaining developmental stability. Coordinated regulation of genes involved in translation and energy production might be important for developmental stability.

## Results

### Expression of *CycG*^*ΔP*^ in wing precursors is sufficient to induce high wing FA

**Fig 1:**
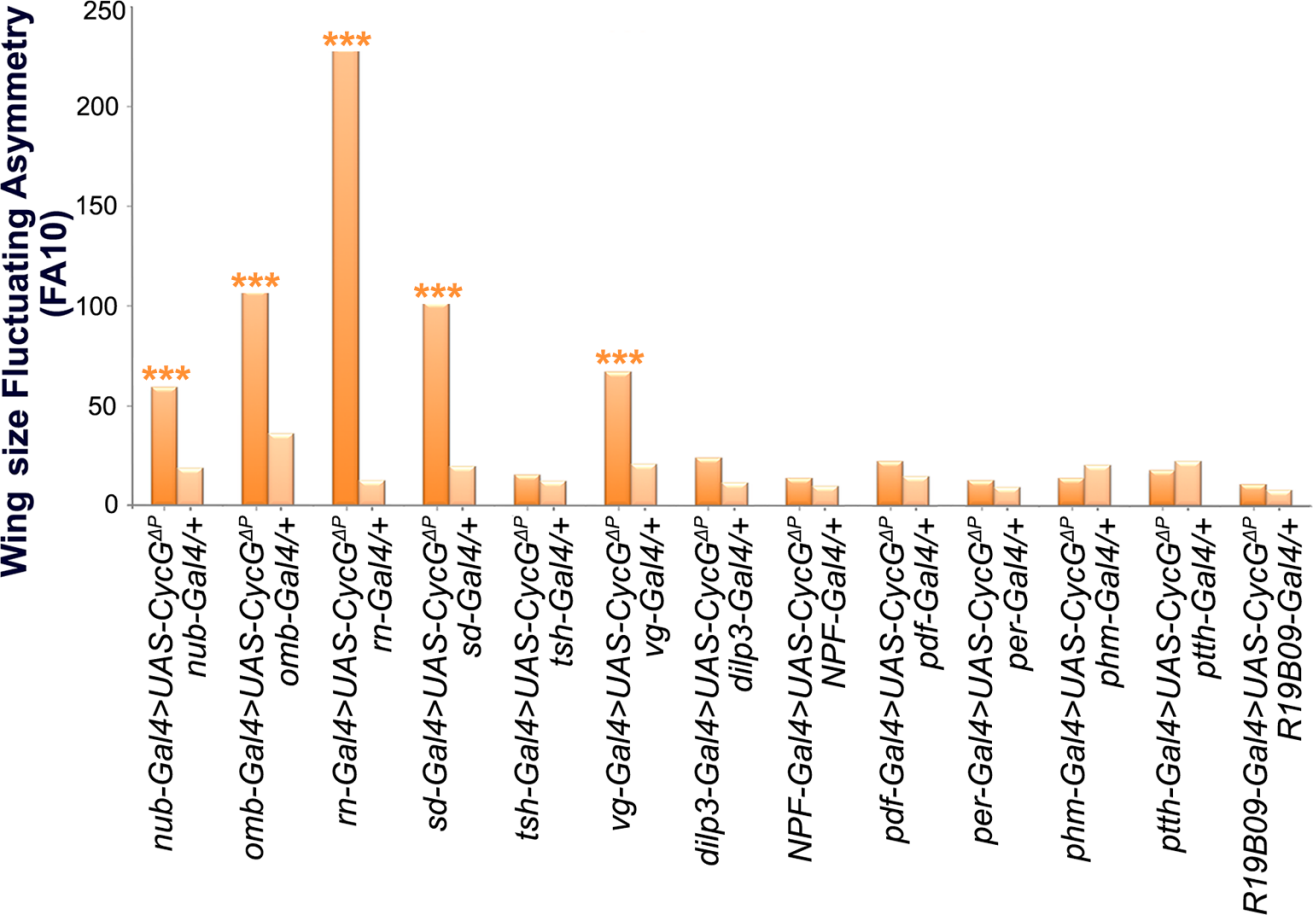
Local deregulation of *CycG* induces high FA. Wing length FA (FA10) of females bearing a Gal4 driver either associated with *UAS-CycG*^*ΔP*^ (dark orange) or alone (light orange). Wing length was measured as the distance between landmarks 3 and 13. (F-tests, *** p-value<0.001, S1 Table). Source data are provided in S2 Table.

We previously reported that expression of *CycG* deleted of the PEST-rich C-terminal domain (amino-acids 541 to 566) (*CycG*^*ΔP*^)—a domain potentially involved in degradation of the protein [26,27]—under control of ubiquitous drivers (*da-Gal4* or *Actin-Gal4*), generated extremely high FA, notably in wings [6]. The strength of this effect was unprecedented in any system or trait. Expression of *CycG*^*ΔP*^ thus provides a unique tool to investigate developmental stability in depth. To determine whether wing FA was due to local or systemic expression of *CycG*^*ΔP*^, we tested a panel of Gal4 drivers specific for wing imaginal discs or neurons. A brain circuit relaying information for bilateral growth synchronization was recently identified [22]. It notably involves a pair of neurons expressing the dILP8 receptor Lgr3 that connects with the insulin-producing cells (IPCs) and the prothoracicotropic hormone (PTTH) neurons. This circuit was particularly appropriate to test the existence of a remote effect of *CycG*^*ΔP*^ expression in generating high FA in the wing. Expression of *CycG*^*ΔP*^ in this neuronal circuit (using *dilp3-*, *NPF-*, *pdf-*, *per-*, *phm-*, *ptth* and *R19B09-Gal4* drivers) did not increase FA of adult wings (Fig 1 and S1 and S2 Tables). Furthermore, expression of *CycG*^*ΔP*^ in cells of the future wing hinge using the *ts-Gal4* driver did not affect wing FA either. By contrast, expressing *CycG*^*ΔP*^ with 5 different wing pouch drivers (*nub-*, *omb-*, *rn-*, *sd-* and *vg-Gal4*) induced high wing FA. We thus concluded that *CycG*^*ΔP*^-induced wing FA was due to an intrinsic response of the growing wing tissue.

### The Cyclin G ETP interacting domain sustains developmental stability

**Fig 2:**
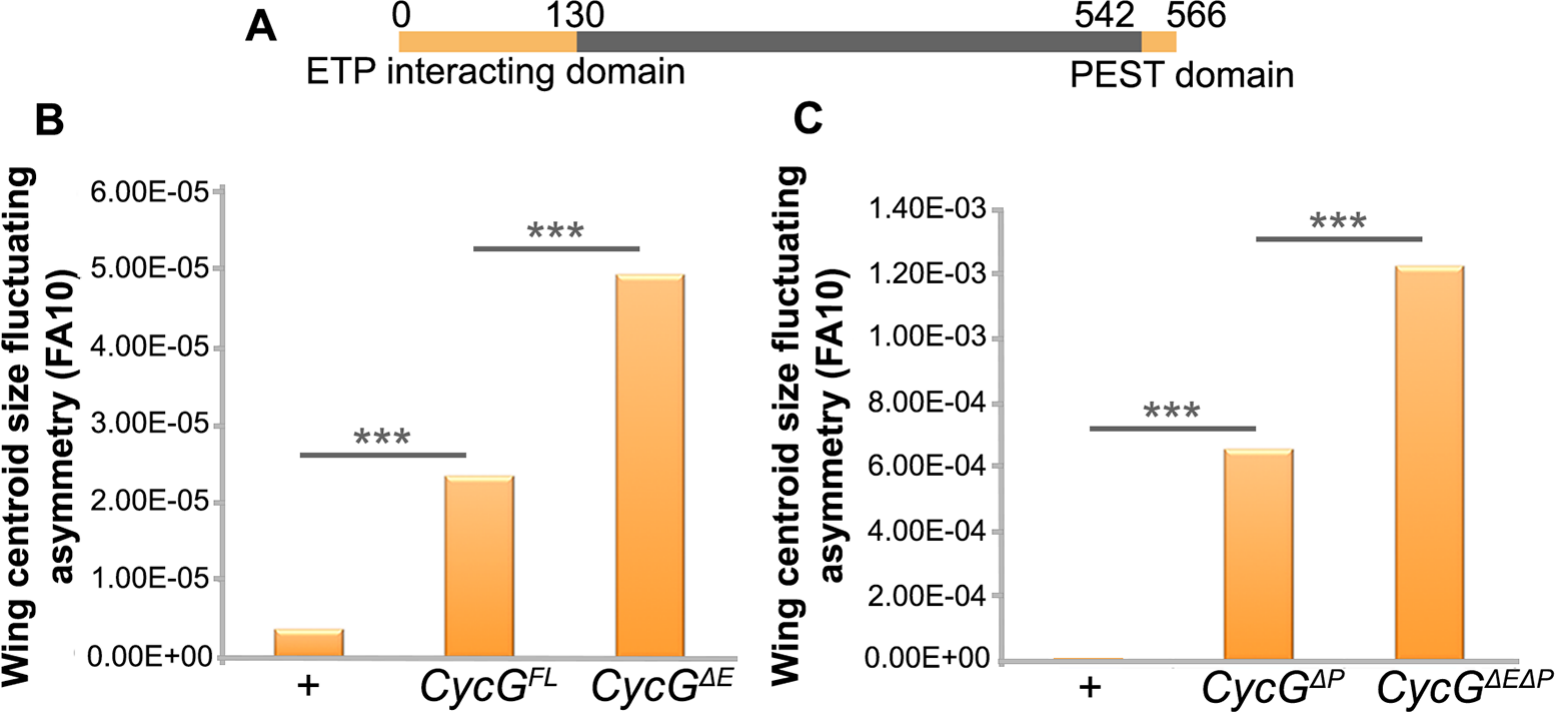
The ETP interacting domain limits *CycG*-induced FA. **A–**Map of the 566 amino-acid Cyclin G protein showing the ETP interacting and PEST domains. **B–**Wing centroid size FA (FA10) of females *da-Gal4*/+ (+), *+/UAS-CycG*^*FL*^*; da-Gal4/+*, (*CycG*^*FL*^) and *+/UAS-CycG*^*ΔE*^; *da-Gal4*, (*CycG*^*ΔE*^). **C–**Wing centroid size FA (FA10) of females *da-Gal4*/+ (+), *+/ UAS-CycG*^*ΔP*^*; da-Gal4/+* (*CycG*^*ΔP*^) and *+/UAS-CycG*^*ΔEΔP*^*; da-Gal4/+* (*CycG*^*ΔEΔP*^). (F-tests, *** p-value<0.001, S3 Table). Source data are provided in S4 Table.

The 566 amino-acid Cyclin G protein exhibits three structured domains: the ETP-interacting domain (amino-acids 1 to 130) that physically interacts with the ETPs Corto and Asx, a cyclin domain (amino-acids 287 to 360) that presents high similarity with the cyclin domain of vertebrate G-type cyclins, and a PEST-rich domain (amino-acids 541 to 566) [28,31]. To test whether the interaction with ETPs, and thus transcriptional regulation by Cyclin G, could be important to control FA, we generated new transgenic lines enabling to express different versions of the *CycG* cDNA: *CycG*^*FL*^ (encoding the full-length protein), *CycG*^*ΔE*^ (encoding an ETP-interacting domain deleted protein), *CycG*^*ΔP*^ (encoding a PEST domain deleted protein), and *CycG*^*ΔEΔP*^ (encoding an ETP-interacting plus PEST domain deleted protein) (Fig 2A). In order to compare the amounts of FA induced, all transgenes were integrated at the same site using the *PhiC31* integrase system. Globally, the different fusion proteins were expressed at the same level (S1 Fig). Contrarily to *da-Gal4*, the wing drivers used above induced not only high FA but also few ectopic veins or small notches that prevented to accurately measure wing centroid size. We then used *da-Gal4* to ubiquitously drive expression of the transgenic lines and focus on the FA phenotype. We confirmed that expression of *CycG*^*ΔP*^ induced very high FA as compared to *+* and *da-Gal4/+* controls. Furthermore, expression of *CycG*^*FL*^ also significantly increased FA, although to a much lesser extent. Interestingly, expression of either *CycG*^*ΔE*^ or *CycG*^*ΔEΔP*^ significantly increased FA as compared to *CycG*^*FL*^ or *CycG*^*ΔP*^, respectively (Fig 2B and 2C, S3 and S4 Tables). These results show that the ETP interacting domain tends to limit *CycG*^*ΔP*^-induced FA and suggest that the interaction between Cyclin G and chromatin regulators sustains developmental stability.

### *CycG* and *PcG* or *ETP* genes interact for developmental stability

**Fig 3:**
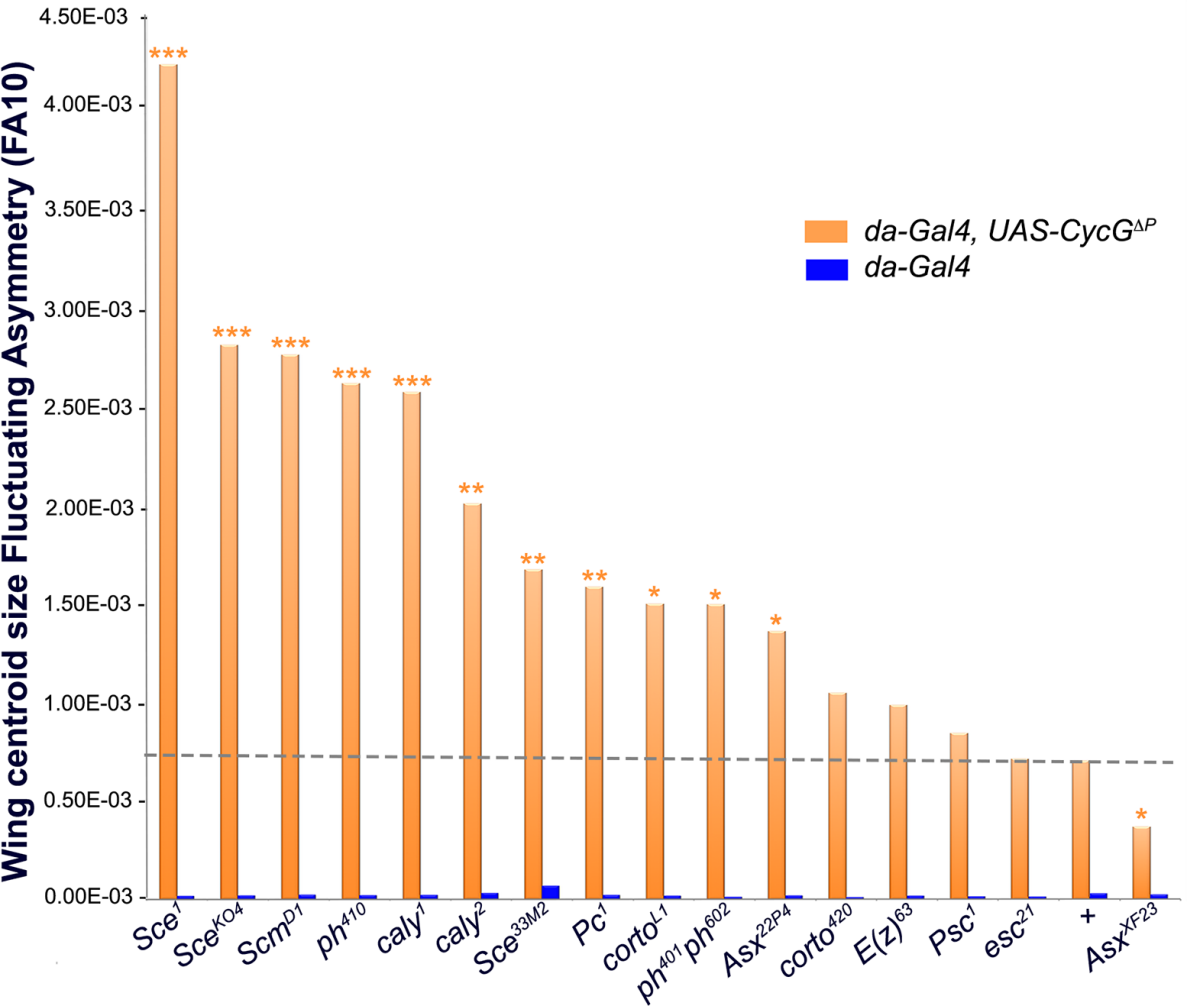
*CycG* interacts with several *PcG* and *ETP* genes for developmental stability. Centroid size FA (FA10) of *ETP* or *PcG* heterozygous mutant females combined with *da-Gal4*, *UAS-CycG*^*ΔP*^ (dark orange; *da-Gal4*, *UAS-CycG*^*ΔP*^; *PcG/+* or *da-Gal4*, *UAS-CycG*^*ΔP*^; *ETP/+*) and *ETP* or *PcG* heterozygous mutant females combined with *da-Gal4* (blue; *da-Gal4/+; PcG/+* or *da-Gal4*; *ETP/+*). The grey dashed line shows FA of *da-Gal4*, *UAS-CycG*^*ΔP*^*/+* females. (F-tests, *p-value<0.05; ** p-value<0.01; *** p-value<0.001, S5 Table). Source data are provided in S6 Table.

We next addressed genetic interactions between *CycG* and *PcG* or *ETP* alleles (Table 1) for developmental stability. FA of flies heterozygous for *PcG* and *ETP* loss of function alleles was not significantly different from that of control flies. However, when combined with *da-Gal4*, *UAS-CycG*^*ΔP*^, many of these mutations significantly increased wing FA as compared to *da-Gal4*, *UAS-CycG*^*ΔP*^ flies (Fig 3, S5 and S6 Tables). This was the case for alleles of the PRC1 and PR-DUB encoding genes *Sex comb extra* (*Sce*^*1*^, *Sce*^*33M2*^ and *Sce*^*KO4*^), *calypso* (*caly*^*1*^ and *caly*^*2*^), *Sex comb on midleg* (*Scm*^*D1*^), *Polycomb* (*Pc*^*1*^), and *polyhomeotic* (*ph-p*^*410*^ and *ph-d*^*401*^*ph-p*^*602*^). No modification of *CycG*^*ΔP*^-induced FA was observed with the *Psc*^*1*^ allele. However, this allele has been described as a complex mutation with loss and gain of function features [32]. Opposite effects were observed for alleles of the ETPs *Asx* and *corto*. *Asx*^*22P4*^ increased *CycG*^*ΔP*^ FA whereas *Asx*^*XF23*^ decreased it. *Asx*^*XF23*^ behaves as a null allele but has not been molecularly characterized [33], whereas the *Asx*^*22P4*^ allele does not produce any protein and thus reflects the effect of ASX loss [34]. Similarly, the *corto*^*L1*^ allele increased *CycG*^*ΔP*^-induced FA whereas the *corto*^*420*^ allele had no effect. To characterize these alleles, we combined them with the *Df(3R)6-7* deficiency that uncovers the *corto* locus, amplified the region by PCR and sequenced. The *corto*^*420*^ allele corresponds to a substitution of 14,209 nucleotides starting at position -59 upstream of the *corto* Transcriptional Start Site (TSS) by a 30-nucleotide sequence. Hence, this allele does not produce any truncated protein. By contrast, *corto*^*L1*^ corresponds to a C towards T substitution that introduces a stop codon at position +73 downstream the TSS, generating a 24 amino-acid polypeptide. *corto*^*L1*^ might then behave as a dominant-negative mutation. Lastly, no modification of *CycG*^*ΔP*^-induced FA was observed for *E(z)*^*63*^ and *esc*^*21*^.

**Table 1:** Polycomb and Enhancer of Polycomb and Trithorax alleles used in this study.

Class	Gene	Allele	Allele class	Reference
*ETP*	*Additional sex combs*	*Asx*^*22P4*^	no protein detected	Scheuermann et al. 2010 [34]
		*Asx*^*XF23*^	loss of function	Simon et al. 1992 [33]
	*corto*	*corto*^*420*^	loss of function	Salvaing et al. 2008 [28]
		*corto*^*L1*^	amorphic	Salvaing et al. 2008 [28]
*PcG*	*calypso*	*caly*^*1*^	no protein detected	Gaytán de Ayala Alonso et al. 2007 [73]; Scheuermann et al. 2010 [34]
		*caly*^*2*^	no protein detected	Gaytán de Ayala Alonso et al. 2007 [73]; Scheuermann et al. 2010 [34]
	*Enhancer of zeste*	*E(z)*^*63*^	loss of function	Beuchle et al. 2001 [74]
	*extra sexcombs*	*esc*^*21*^	amorphic	Gindhart and Kaufman 1995 [75]
	*Polycomb*	*Pc*^*1*^	amorphic	Capdevila et al. 1986 [76]
	*polyhomeotic*	*ph-d*^*401*^*ph-p*^*602*^	null	Dura et al. 1987 [77]
	*polyhomeotic proximal*	*ph-p*^*410*^	loss of function	Dura et al. 1987 [77]
	*Posterior sex combs*	*Psc*^*1*^	hypomorphic	Adler et al. 1989 [78]
	*Sex combs extra*	*Sce*^*1*^	null	Gorfinkiel et al. 2004 [79]
		*Sce*^*33M2*^	loss of function	Fritsch et al. 2003 [80]
		*Sce*^*KO4*^	null	Gutiérrez et al. 2012 [81]
	*Sex comb on midleg*	*Scm*^*D1*^	amorphic	McKeon and Brock 1991 [82]

Interestingly, *Asx* and *caly* encode proteins of the PR-DUB complex whereas *Pc*, *ph*, *Sce* and *Scm* encode proteins of PRC1, and *E(z)* and *esc* encode proteins of PRC2. Taken together, these results indicate that Cyclin G interacts with the Polycomb complexes PRC1 and PR-DUB, but not with PRC2, for developmental stability.

### Expression of *CycG*^*ΔP*^ or *CycG*^*ΔEΔP*^ does not modify global H2AK118ub

**Fig 4:**
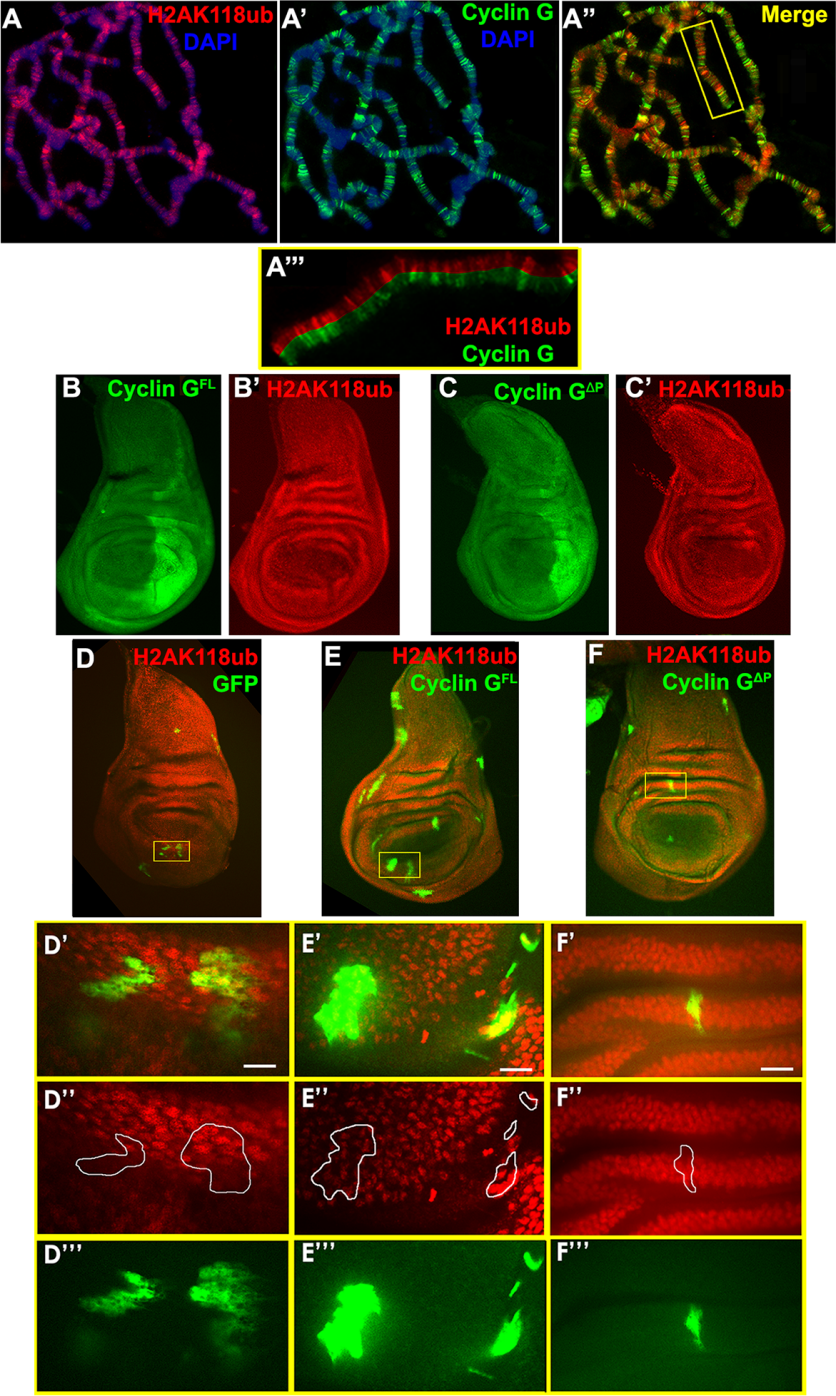
Cyclin G co-localizes with H2AK118ub at many sites on polytene chromosomes but overexpression of *CycG* does not modify global H2AK118ub. **A, A’, A”–**Immunostaining of polytene chromosomes from *w*^*1118*^ third instar larvae. H2AK118ub (red), Cyclin G (green), DAPI (blue). **A”‘**–Close-up of the box showed in A”. **B, B’–**Wing imaginal discs of 3^rd^ instar larvae expressing *CycG*^*ΔP*^ in the posterior compartment under control of the *en-Gal4* driver, stained with anti-Cyclin G (green) and anti-H2AK118ub (red). **C, C’–**Wing imaginal discs of 3^rd^ instar larvae expressing *CycG*^*ΔEΔP*^ in the posterior compartment under control of the *en-Gal4* driver, stained with anti-Cyclin G (green) and anti-H2AK118ub (red). **D, D’, D”, D”’**–GFP clones in wing imaginal discs stained with anti-H2AK118ub (red). D’, D” and D”’ are close-up views of the yellow rectangle shown in D. **E, E’, E”, E”’**–*CycG*^*ΔP*^ clones marked by GFP in wing imaginal discs stained with anti-H2AK118ub (red). E’, E” and E”’ are close-up views of the yellow rectangle shown in E. **F, F’, F”, F”’**–*CycG*^*ΔEΔP*^ clones marked by GFP in wing imaginal discs stained with anti-H2AK118ub (red). F’, F” and F”’ are close-up views of the yellow rectangle shown in F. Scale bars: 50 μm.

Cyclin G binds polytene chromosomes at many sites and co-localizes extensively with Ph and Asx suggesting a potential interaction with PRC1 and PR-DUB on chromatin [28,31]. *Sce* and *caly* encode antagonistic enzymes of the PRC1 and PR-DUB complexes, respectively. Sce, aka dRing, ubiquitinates histone H2A on lysine 118 (H2AK118ub) whereas Calypso, aka dBap1, deubiquitinates the same H2A residue [34,35]. To investigate whether Cyclin G was related to these ubiquitin ligase/deubiquitinase activities, we immunostained polytene chromosomes from *w*^*1118*^ larvae with anti-Cyclin G and anti-human H2AK119ub antibodies (homologous to *Drosophila* H2AK118ub) [36,37]. Cyclin G and H2AK118ub co-localized extensively on chromosome arms suggesting that Cyclin G transcriptional activity might somehow be connected to this histone mark (Fig 4A). Wing imaginal discs presented a uniform pattern of H2AK118ub. When either *CycG*^*ΔP*^ or *CycG*^*ΔEΔP*^ was expressed in the posterior compartment of wing imaginal discs using the *en-Gal4* driver, the global amount of H2AK118ub was not markedly modified (Fig 4B and 4C). Similarly, clones expressing *CycG*^*ΔP*^ or *CycG*^*ΔEΔP*^ showed the same amount of H2AK118ub than control GFP clones (Fig 4D, 4E and 4F). We thus concluded that high FA was not related to a global perturbation of H2AK118 ubiquitination.

### Cyclin G controls the expression of genes involved in translation and energy production

Cyclin G controls transcription of the homeotic gene Abdominal-B and more specifically behaves as an Enhancer of PcG gene for the regulation of homeotic gene expression [31,38]. However, the high number of Cyclin G binding sites on polytene chromosomes suggests that it has many other transcriptional targets. We thus hypothesized that *CycG*^*ΔP*^-induced FA might be related to the deregulation of Cyclin G transcriptional targets. To further address the role of Cyclin G in transcriptional regulation, we deep-sequenced transcripts from *da-Gal4*, *UAS-CycG*^*ΔP*^*/+* wing imaginal discs. Considering that the Gal4 transactivator might unspecifically interfere with transcription of some genes, we deep-sequenced *da-Gal4/+* wing imaginal disc transcripts as negative control.

**Fig 5:**
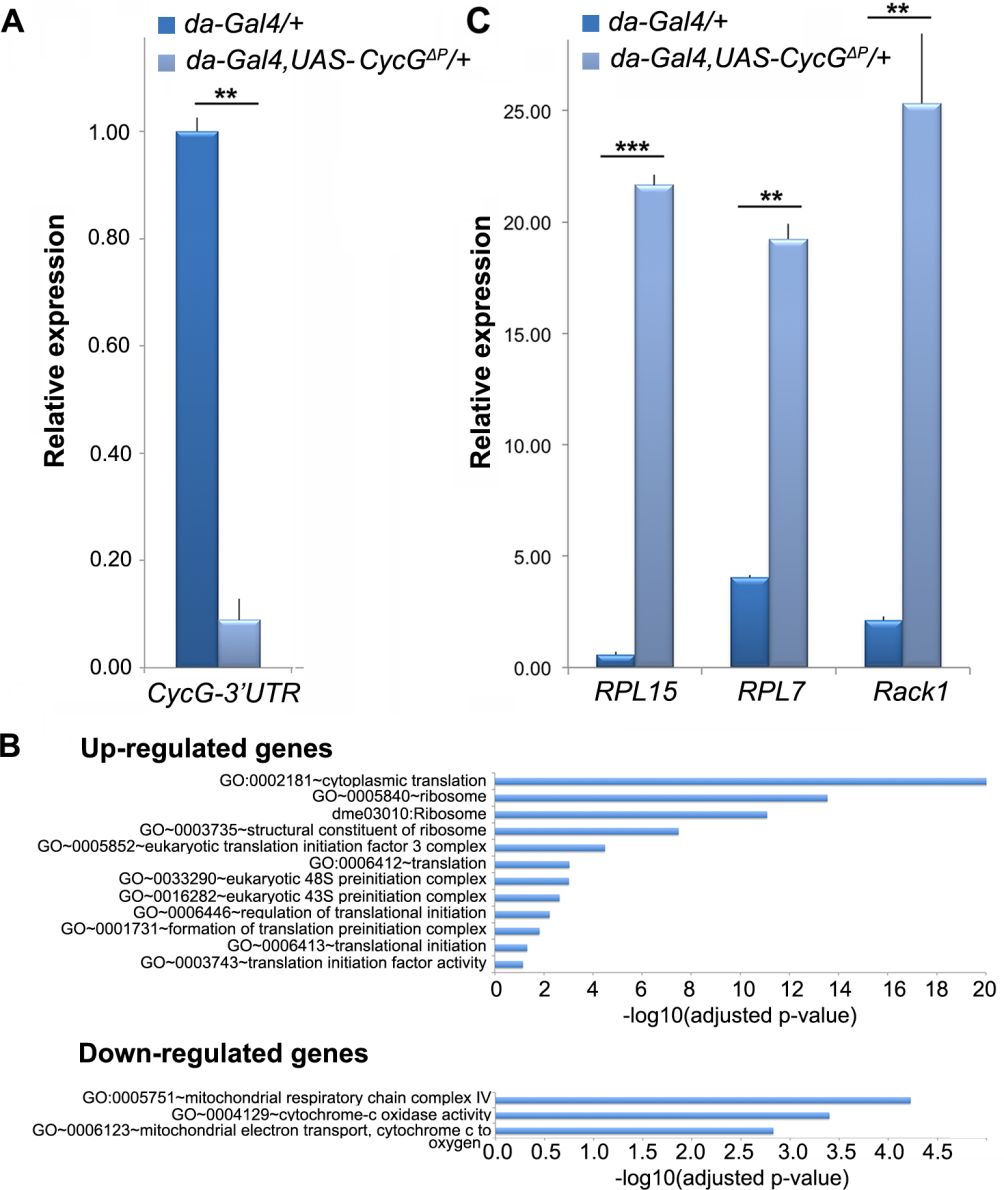
Genes deregulated in wing imaginal discs expressing *CycG*^*ΔP*^. **A–**RT-qPCR analysis of endogenous *CycG* expression in *da-Gal4*,*UAS-CycG*^*ΔP*^*/+* and *da-Gal4/+* wing imaginal discs. Expression of *CycG* was normalized on the geometric mean of *Lam* and *rin* (S8 Table). t-tests, ** p-value<0.01. Error bars correspond to standard deviations. **B–**Ontology of up-regulated and down-regulated genes in *da-Gal4*, *UAS-CycG*^*ΔP*^*/+ vs da-Gal4/+* wing imaginal discs. Gene ontology analysis was performed with DAVID (S9 Table). **C–**RT-qPCR analysis of *RPL15*, *RPL7* and *Rack1* expression in *da-Gal4*, *UAS-CycG*^*ΔP*^*/+* and *da-Gal4/+* wing imaginal discs. Expression of *RPL15*, *RPL7* and *Rack1* were normalized on the geometric mean of *Lam* and *rin* (S10 Table). t-tests, ** p-value<0.01. Error bars correspond to standard deviations. t-tests, ** p-value<0.01; *** p-value<0.001.

Sequence reads were aligned with the *D. melanogaster* genome to generate global gene expression profiles. With an adjusted p-value threshold of 0.05, we retrieved 530 genes whose expression was significantly different between *da-Gal4*, *UAS-CycG*^*ΔP*^*/+* and the *da-Gal4/+* control (S7 Table). Surprisingly, expression of *CycG* was only weakly induced in *da-Gal4*, *UAS-CycG*^*ΔP*^*/+* imaginal discs (1.3 fold). To test the hypothesis that Cyclin G could, directly or not, regulate its own repression, we quantified expression of the endogenous *CycG* gene by RT-qPCR using primers located in the 3’UTR that were not present in the transgene. Indeed, expression of endogenous *CycG* was significantly decreased upon *CycG*^*ΔP*^ induction, suggesting that Cyclin G repressed its own expression (Fig 5A and S8 Table). Among the 530 genes deregulated in *da-Gal4*, *UAS-CycG*^*ΔP*^*/+* imaginal discs, 216 were up-regulated and 314 down-regulated. Up-regulated genes were enriched in the Gene Ontology categories *cytoplasmic translation* and *translational initiation* whereas down-regulated genes were enriched in the category *mitochondrial respiratory chain complex* (Fig 5B and S9 Table). By RT-qPCR, we verified that several ribosomal protein genes (*RpL15*, *RpL7* and *Rack1*) were over-expressed in *da-Gal4*, *UAS-CycG*^*ΔP*^*/+* imaginal discs (Fig 5C and S10 Table). In conclusion, *CycG*^*ΔP*^*-*induced FA correlates with activation of genes involved in translation and repression of genes involved in energy production.

### Cyclin G binds the TSS of genes involved in translation and protein phosphorylation

**Fig 6:**
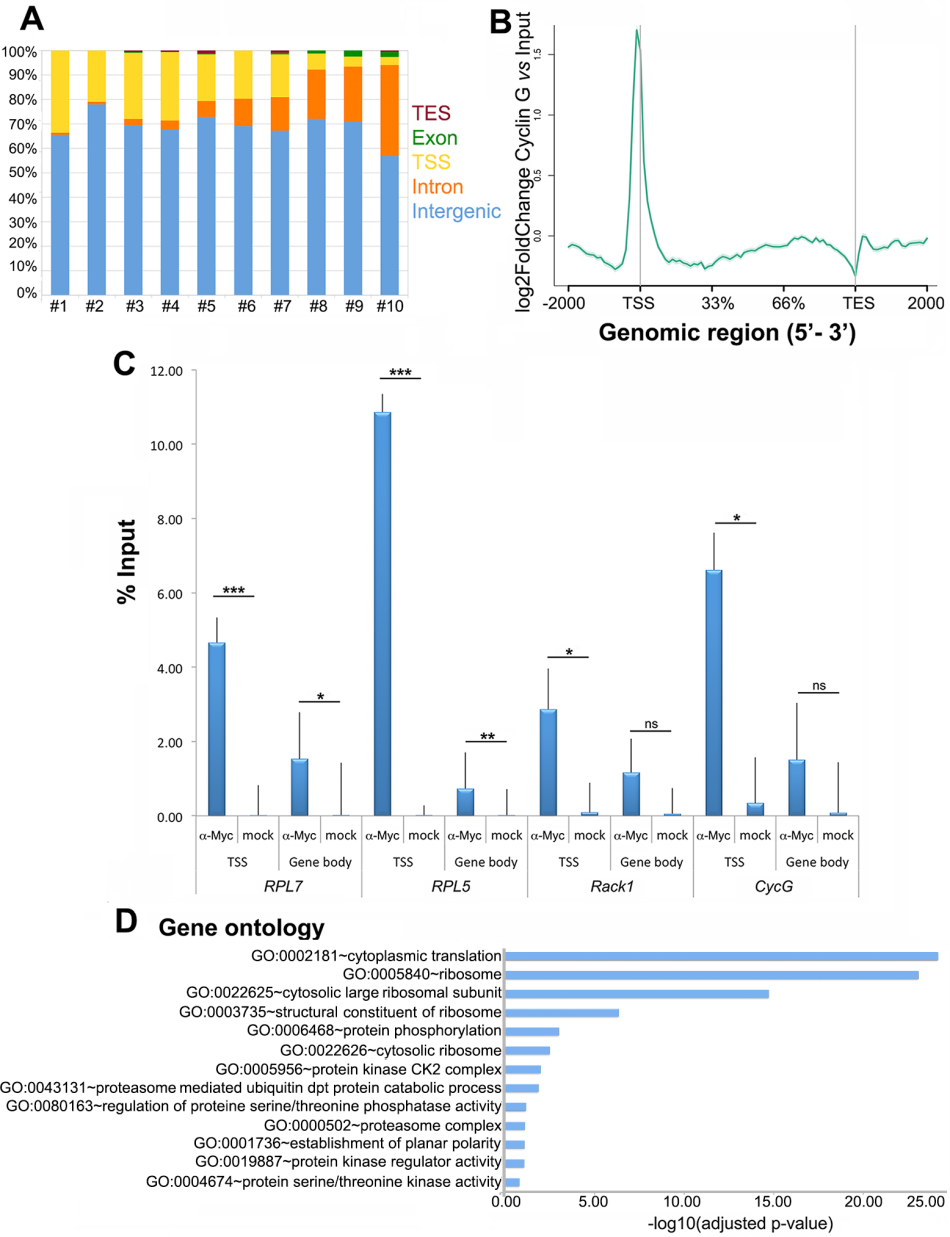
Identification of Cyclin G genome-wide binding sites in wing imaginal discs. **A–**Repartition of feature types among the 3363 decile-ranked Cyclin G peaks (S12 Table). Validated peaks were ranked based on their height (highest number of overlapping reads), and separated in ten bins before annotation. **B–**Average profile of Cyclin G signal over these genes shown as an aggregation plot. The standard error is represented as a shaded area over the curve. **C–**ChIP-qPCR analysis of *RPL7*, *RPL5*, *Rack1* and *CycG*. IPs were performed either with Myc antibody (α-Myc) to reveal the presence of Cyclin G, or with rabbit IgG as negative control (mock). qPCR were performed using oligonucleotide primers located either at the TSS or in the gene body as indicated. Error bars represent the coefficient of variation (CV) (S13 Table). **D–**Ontology of the 889 genes. Gene ontology analysis was performed with DAVID.

**Fig 7:**
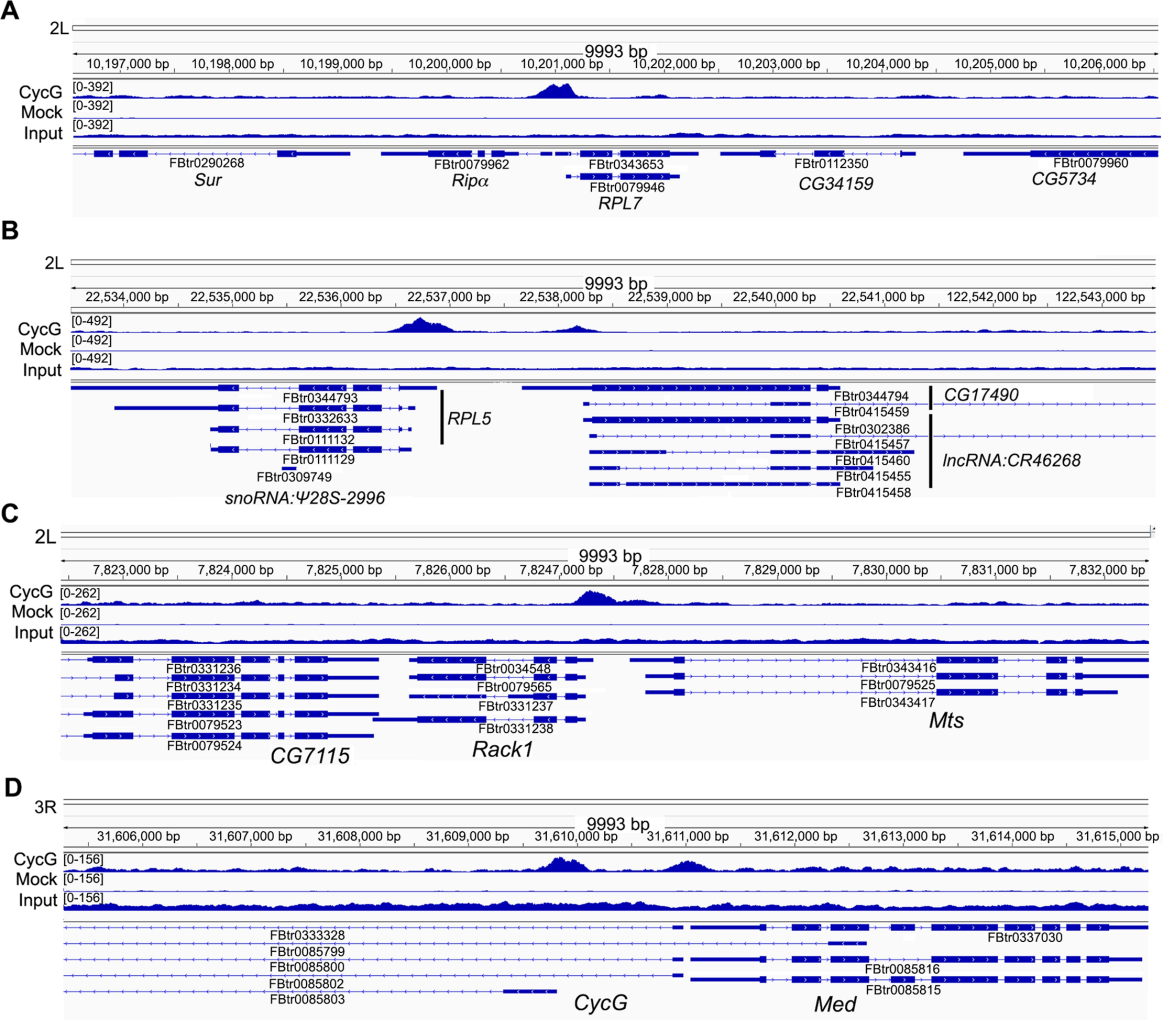
Snapshots illustrated Cyclin G ChIP-seq. Focus on *RPL7* (A), *RPL5* (B), *Rack1* (C) and *CycG* (D).

We next sought to determine the direct transcriptional targets of Cyclin G by ChIP-seq. To do this, we took advantage of the transgenic *+/ UAS-CycG*^*ΔP*^ line in which Cyclin G was fused to a Myc tag. We performed ChIP experiments with anti-Myc antibodies using chromatin from *+/ UAS-CycG*^*ΔP*^*; da-Gal4/+* wing imaginal discs or *da-Gal4/+* wing imaginal discs (mock ChIP). 3363 significant peaks were identified (IDR < 0.05) in *+/ UAS-CycG*^*ΔP*^*; da-Gal4/+* wing imaginal discs. Among these peaks, 1045 were located on a subset of 889 genes, most of them corresponding to TSS (Fig 6A and 6B, and S11 and S12 Tables). We could not formally exclude that there were differences in Myc-Cyclin G binding and endogenous Cyclin G binding. However, the increase in *CycG* mRNA being low (1.3 fold), we assumed that the midly over-expressed exogenous *CycG*^*ΔP*^ would respect the binding pattern of the endogenous protein. Snapshots of some TSS bound genes (*RPL7*, *RPL5*, *Rack1*, *CycG*) are shown on Fig 7. ChIP-qPCR analysis of these four genes confirmed that Cyclin G peaked on their TSS and decreased on gene body (Fig 6C and S13 Table). As endogenous *CycG* was down-regulated when *CycG*^*ΔP*^ was expressed and Cyclin G bound its own TSS, this confirmed that Cyclin G represses its own promoter.

The 889 Cyclin G-bound genes were enriched in GO categories *cytoplasmic translation* and *protein phosphorylation* (Fig 6D). Comparison of the 530 genes deregulated in wing imaginal discs expressing *CycG*^*ΔP*^ with these 889 genes showed that only 62 genes were both deregulated (39 up- and 23 down-regulated) and bound by Cyclin G (S14 Table), suggesting that most of the effects on gene expression were indirect. Strikingly, the 39 up-regulated genes were significantly enriched in the GO category *translation* (GO:0002181~cytoplasmic translation, 14 genes, enrichment score: 12.31, adjusted p-value 2.07E-16) and the 23 down-regulated ones in the GO category *cytochrome-c oxidase activity* (GO:0004129~cytochrome-c oxidase activity, 3 genes, enrichment score: 2.01, adjusted p-value 4.40E-02).

### Cyclin G-bound genes are enriched in PRC1 and Asx

**Fig 8:**
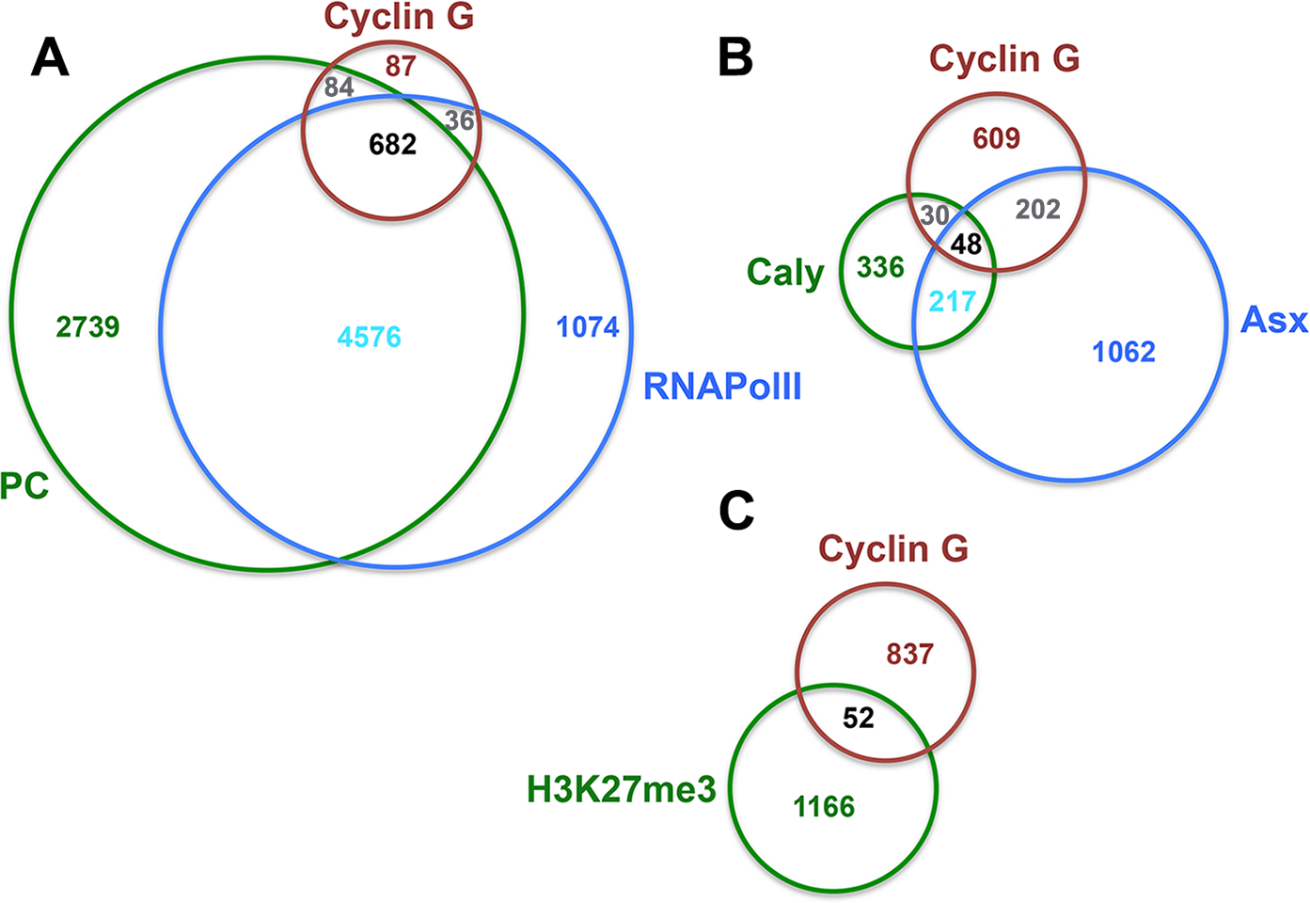
Cyclin G shares target genes with PRC1, Asx and RNAPolII but not with Calypso. Venn diagrams showing the intersection between Cyclin G-bound genes in *+/ UAS-CycG*^*ΔP*^*; da-Gal4/+* wing imaginal discs with Pc and RNAPlII (A), Asx and Calypso (B), and K3K27me3 (C) in wild-type wing imaginal discs.

Using published datasets, we analysed the correlation between regions bound by Cyclin G in +/*UAS-CycG*^*ΔP*^*; da-Gal/+* imaginal discs and those enriched in PRC1, or PR-DUB components, RNAPolII, or H3K27me3 in wild type wing imaginal discs (S15 Table). Cyclin G-bound regions were significantly exclusive from H3K27me3, corroborating previous results [31]. The same comparisons were performed gene-wise and gave the same results (Fig 8). Importantly, 78% of Cyclin G-bound genes were also bound by RNAPolII and Pc. We cannot exclude that Cyclin G might co-localize with PRC1 in some cells and with RNAPolII in others. Alternatively, Cyclin G-bound genes might be simultaneously bound by PRC1 and RNAPolII. Considering RNAPolII as a proxy for transcriptional activity, and given that PRC1 has the ability to block transcriptional initiation [39], these genes would be most probably paused. Cyclin G also shared many target genes with Asx but, though Asx and Calypso belong to the PR-DUB complex, Cyclin G did not share binding sites with Calypso. This indicates either that the interaction between Cyclin G and Asx destabilizes the PR-DUB complex or that it takes place outside PR-DUB.

### Cyclin G is a hub in the wing imaginal disc network

These genome-wide analyses indicate that Cyclin G coordinates the expression of genes involved in translation and energy production. However, only a few Cyclin G-bound genes were deregulated in *da-Gal4*, *UAS-CycG*^*ΔP*^*/+* imaginal discs. To better understand how Cyclin G orchestrates target gene expression, we developed a systems biology approach. We first built an interactome based on genes expressed in control *da-Gal4/+* wing imaginal discs (with a cutoff of 10 reads). Edges corresponding to protein-protein interactions (PPI) and transcription factor-gene interactions (PDI) were integrated into this interactome through DroID [40]. The resulting wing imaginal disc interactome, further called the WID network, was composed of 9,966 nodes (proteins or genes) connected *via* 56,133 edges (interactions) (WID.xmml). We then examined the position of Cyclin G in this network. Betweenness centrality—*i*.*e*. the total number of non-redundant shortest paths going through a certain node–is a measure of centrality in a network [41]. A node with a high betweenness centrality could control the flow of information across the network [42]. With 8.32E-03, Cyclin G had one of the highest value of betweenness centrality of the network, ranking at the 30^th^ position among the 9,966 nodes. This suggests that Cyclin G represents a hub in the WID network.

**Fig 9:**
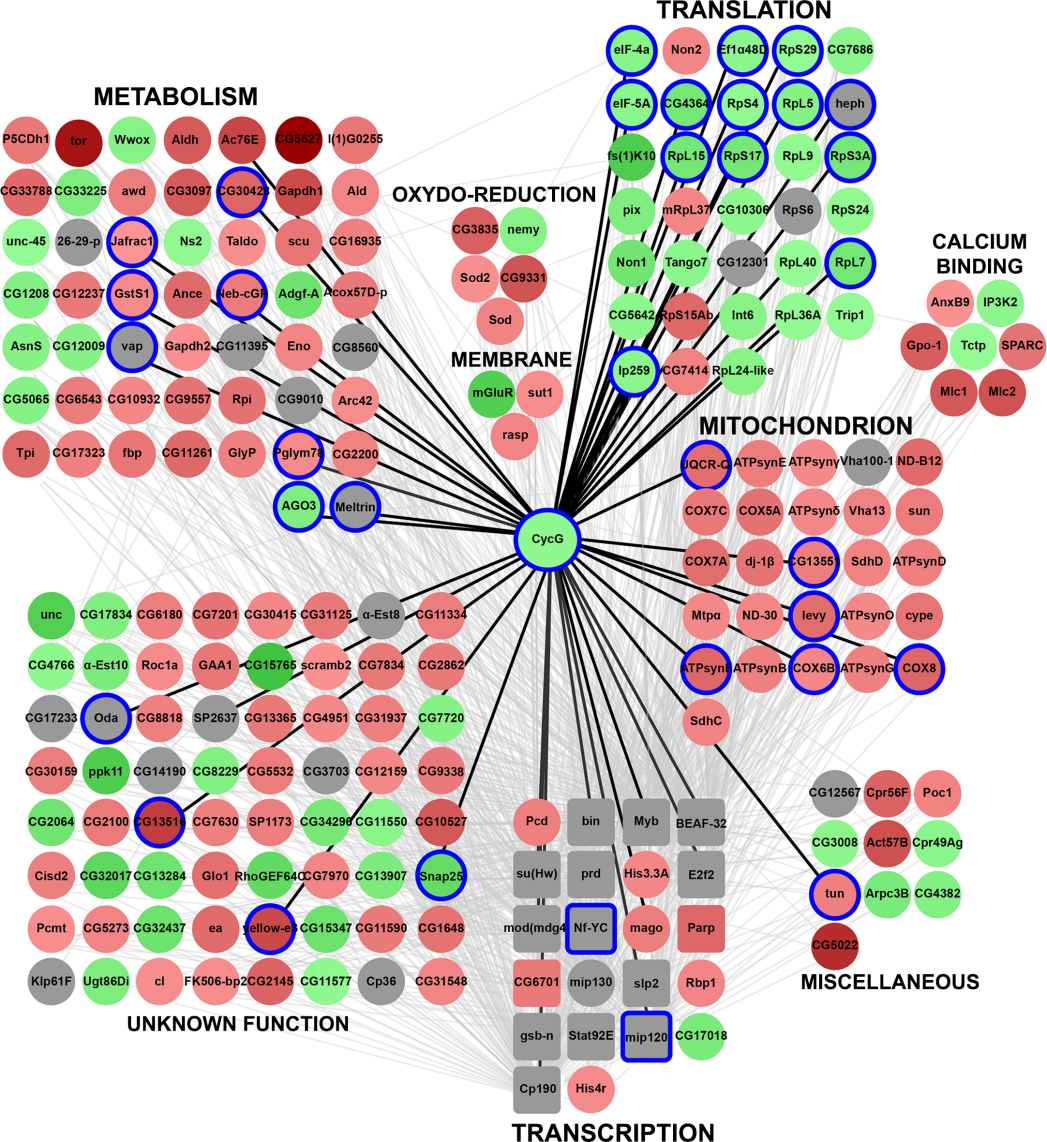
Functional subnetwork identified in wing imaginal discs expressing *CycG*^*ΔP*^. Schematic representation of a sub-network of 222 genes centred on Cyclin G (CycG_subnetwork.xmml) and identified using JactiveModules (Z score 48.53). In this sub-network, 65 genes were up-regulated in *da-Gal4*, *UAS-CycG*^*ΔP*^ *vs da-Gal4/+* wing imaginal discs (green gradient), 124 genes were down-regulated (red gradient), and 33 genes were not significantly deregulated (grey). Genes bound by Cyclin G are circled in blue. Transcription factor genes are represented by squares. Genes were clustered depending on their function. Black edges correspond to interactions discovered in the present study. Grey edges correspond to interactions described in the literature and imported into the WID network using DroID.

**Fig 10:**
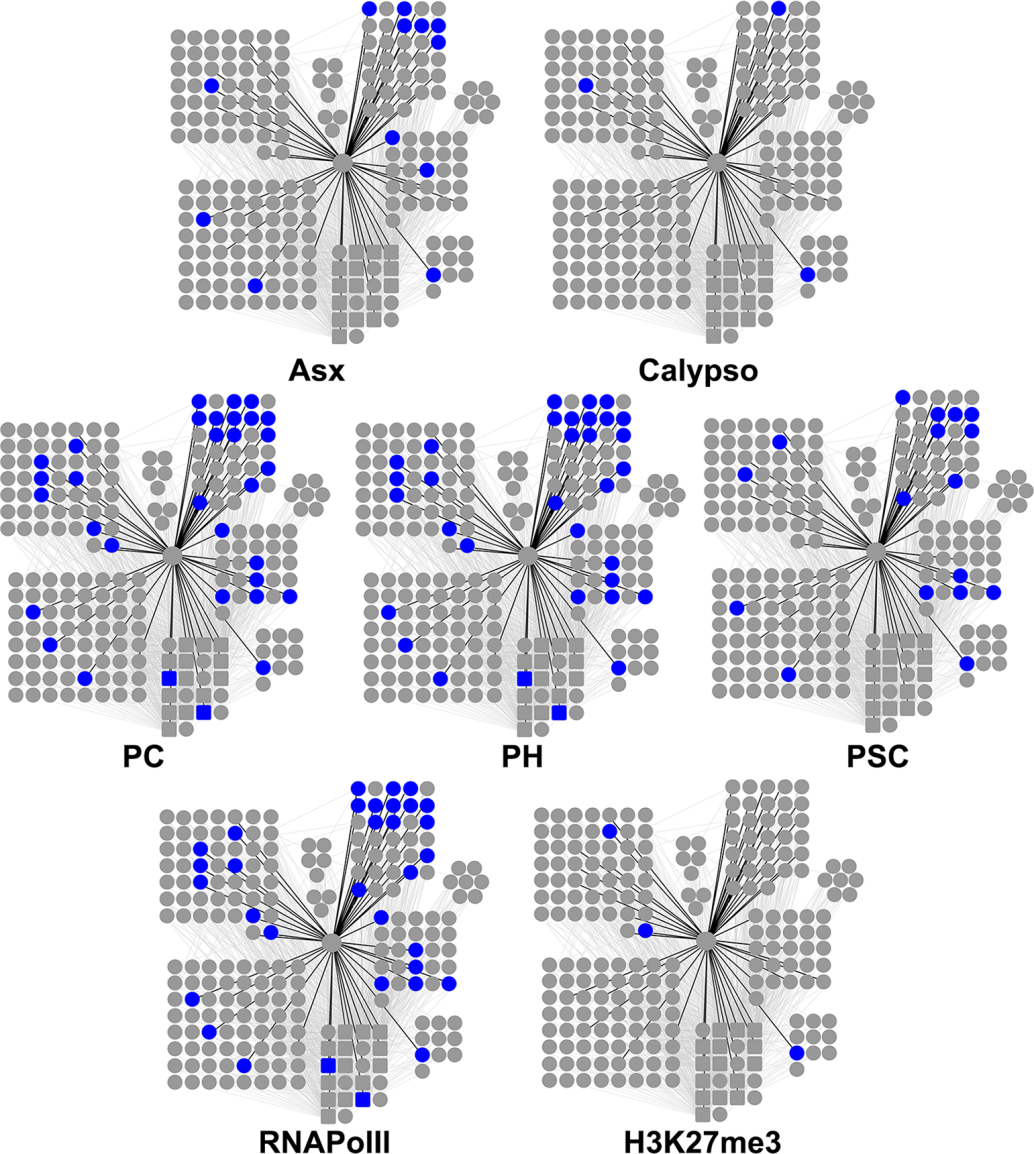
Genes bound by Asx, Calypso, Pc, Ph, Psc, or RNAPolII, or enriched in H3K27me3 in the sub-network of 222 genes centred on Cyclin G. Bound genes are represented in blue.

In order to isolate a connected component of the WID network that showed significant expression change when *CycG*^*ΔP*^ is expressed, we introduced the expression matrix describing expression of the 530 significantly deregulated genes in the WID network. We next used JactiveModules to identify sub-networks of co-deregulated genes [43]. A significant sub-network of 222 nodes and 1069 edges centred on Cyclin G was isolated (Z score 48.53). This sub-network was laid out according to functional categories (Fig 9, CycG_subnetwork.xmml). Four modules composed of genes respectively involved in transcription, mitochondrial activity, translation, and metabolism, were found to be highly connected to Cyclin G. Strikingly, the “translation” module was mainly composed of genes up-regulated in *da-Gal4*, *UAS-CycG*^*ΔP*^*/+* wing imaginal discs. On the contrary, the “mitochondrion” and “metabolism” modules were mainly composed of genes down-regulated in *da-Gal4*, *UAS-CycG*^*ΔP*^*/+* wing imaginal discs. Interestingly, Cyclin G-bound genes in this sub-network were enriched in genes bound by the PRC1 proteins Pc, Ph and Psc, as well as by RNAPolII, and to a lesser extent by Asx (Fig 10).

## Discussion

The *CycG* gene of *D*. *melanogaster* encodes a cyclin involved in transcriptional control, cell growth and the cell cycle [26,28,38]. Mild overexpression of Cyclin G induces high fluctuating asymmetry (FA), notably of wings, providing a unique tool to investigate the genetic bases of developmental stability [6,7]. Cyclin G interacts physically with two chromatin regulators of the Enhancers of Trithorax and Polycomb family (ETP), and genetically with *Polycomb-group* (*PcG*) genes [31]. This prompted us to examine the role of these interactions in developmental stability and to investigate deeply the function of Cyclin G in transcriptional regulation.

### Cyclin G maintains developmental stability through an organ-autonomous process that involves the PRC1 and PR-DUB complexes

*CycG-*induced wing FA only occured when the deregulation was local, *i*.*e*. in wing imaginal discs. Although we cannot exclude that Cyclin G induces expression of a systemic factor that is released into the haemolymph, our observations suggest that *CycG* maintains developmental stability through an organ-autonomous mechanism which would not involve the *Dilp8/Lgr3* pathway. Many Cyclin G targets in the wing imaginal discs are also bound by PRC1, by Asx and by RNAPolII, but are not enriched in H3K27me3. In agreement, mutations in PRC1 and PR-DUB, but not in PRC2, strongly increase *CycG-*induced FA. We did not observe any significant overlap between Cyclin G-bound genes and binding sites for Calypso, the second component of PR-DUB. Yet, *caly* mutations also strongly increase *CycG*-induced FA. Thus, the role of PR-DUB in this context remains to be clarified. PRC1 and PR-DUB contain antagonistic enzymes (Sce/dRing and Calypso) that respectively ubiquitinates and deubiquitinates H2AK118. However, the global level of H2AK118 ubiquitination is not modified in tissues where Cyclin G isoforms are overexpressed suggesting that this epigenetic mark is not involved in developmental stability. Cyclin G targets strikingly remind the neo-PRC1 targets described in [44]. Indeed, PRC1 components are redeployed during development and control these neo-PRC1 targets that are robustly transcribed, not enriched in H2AK118ub, and on which PRC1 is recruited independently of PRC2 [44]. It was proposed that PRC1 limits the expression of these neo-PRC1 genes that are mainly involved in cell proliferation, cell signaling and polarity, thus explaining its tumor suppressor role [44]. Hence, Cyclin G might participate with PRC1 and PR-DUB in the control of these neo-PRC1 genes and this might be important for developmental stability.

### Deregulation of genes involved in translation, metabolism and mitochondrial activity correlates with perturbation of developmental stability

*Drosophila* Cyclin G and the two vertebrate G-type cyclins, CCNG1 and CCNG2 exhibit a complex relationship to growth, on the one hand promoting it, [45–48] and on the other hand, slowing down or even blocking the cell cycle [26,49–52]. Accordingly, we found that Cyclin G controls a small regulatory sub-network connecting genes involved in metabolism, mitochondrial activity and translation. Notably, many genes involved in basic metabolism, such as *Gapdh1*, *Gapdh2* or *Jafrac1*, are down-regulated in the *CycG*^*ΔP*^ context, which also agrees with the small mean size of *CycG*^*ΔP*^ flies, organs and cells. A large scale analysis of the *Drosophila* wing imaginal disc proteome has recently shown that wing size correlates with some basic metabolic functions, positively with glucose metabolism and negatively with mitochondrial activity, but not with ribosome biogenesis [53]. However, in *CycG*^*ΔP*^ flies while mitochondrial genes are negatively regulated, ribosomal biogenesis genes are simultaneously positively regulated. Although transcriptome variations are probably not a direct image of proteome variations, our data suggest that robustness of wing size correlates with the fine-tuning of these key functions relative to each other. Corroborating our study, a mutant for the AAA mitochondrial protease FTSH4 in *Arabidospsis thaliana* displays high variability of sepal size and shape associated with early ROS production [54]. Furthermore, a recent analysis of human mesothelioma cells also points to a role of BAP1 and the PR-DUB complex in mitochondrial function and ROS homeostasis [55]. It will be important in the future to understand how epigenetic control of genes involved in mitochondrial activity and control of growth impact developmental stability and how deregulation of these processes might lead to cancer.

## Materials and methods

### Plasmids

The *pPMW-attB* plasmid was built as follows: the Gateway vector *pPMW* [56] was linearized by digestion with *NsiI*; the *attB* sequence was amplified from *pUASTattB* [57] (using primers *attB-NsiIF* and *attB-NsiIR* (S16 Table) and the PCR product was digested with *NsiI*; the digested PCR product and the linearized plasmid were ligated and sequenced. This plasmid was deposited at Addgene (plasmid # 61814).

The full-length *CycG* cDNA (*CycG*^*FL*^, encoding the 566 amino-acid protein) was amplified from S2 cell cDNAs using primers *CycGnF* and *CycGnR*. cDNAs encoding truncated forms of Cyclin G (*CycG*^*ΔP*^, Cyclin G deleted of the putative PEST domain corresponding to amino-acids 542 to 566; *CycG*^*ΔE*^, Cyclin G deleted of the ETP-interacting domain corresponding to amino-acids 1 to 130; *CycG*^*ΔEΔP*^, Cyclin G deleted of both domains) were amplified from the full-length *CycG* cDNA using primers *CycGnF* and *CycG541R*, *CycG130F* and *CycGnR*, and *CycG130F* and *CycG541R*, respectively (S16 Table). The PCR products were cloned into *pENTR/D-TOPO* (Invitrogen), transferred into *pPMW-attB* and the resulting plasmids *pPMW-attB-CycG*^*FL*^, *pPMW-attB-CycG*^*ΔP*^, *pPMW-attB-CycG*^*ΔE*^, *pPMW-attB-CycG*^*ΔEΔP*^ were sequenced.

### *Drosophila melanogaster* strains and genetics

Flies were raised on standard yeast-cornmeal medium at 25°C.

*UAS-Myc-CycG* transgenic lines were obtained by *PhiC31*-integrase mediated insertion into strain *y*^*1*^*M{vas-int*.Dm}ZH-2Aw^*^;M{3xP3-RFP.*attP'}ZH-51C* (stock BL-24482). Plasmids *pPMW-attB-CycG*^*FL*^, *pPMW-attB-CycG*^*ΔP*^, *pPMW-attB-CycG*^*ΔE*^ and *pPMW-attB-CycG*^*ΔEΔP*^ were injected into embryos, G0 adults were back-crossed to *yw*, and G1 transformants were crossed to *yw* again to obtain G2 transformants (BestGene Inc.). Transformants were individually crossed with *yw; Sp/CyO*, and the curly wing siblings were crossed with each other. Homozygous transgenic lines were then obtained by crossing 5 females and 5 males. The resulting lines were named *UAS-CycG*^*FL*^, *UAS -CycG*^*ΔP*^, *UAS-CycG*^*ΔE*^ and *UAS -CycG*^*ΔEΔP*^.

Gal4 drivers used were *daughterless-Gal4* (*da-Gal4*), *engrailed-Gal4* (*en-Gal4*, *nubbin-Gal4* (*nub-Gal4*), *optomotor-blind-Gal4* (*omb-Gal4*), *rotund-Gal4* (*rn-Gal4*), *scalloped-Gal4* (*sd-Gal4*), *teashirt-Gal4* (*tsh-Gal4*), *vestigial-Gal4* (*vg-Gal4*) (from the Bloomington *Drosophila* stock center), and *Insulin-like peptide 3-Gal4* (*dILP3-Gal4*), *neuropeptide F-Gal4* (*NPF-Gal4)*, *Pigment-dispersing factor-Gal4* (*Pdf-Gal4*), *period-Gal4* (*per-Gal4*), *phantom-Gal4* (*phm-Gal4*), *Prothoracicotropic hormone-Gal4* (*Ptth-Gal4*), *R10B09-Gal4* (kind gifts from Dr M. Dominguez’s lab).

To generate clones, the strain *hs-flp; tub>stop>Gal4*, *UAS-GFP/CyO* (a kind gift of Dr. M. Gho) was crossed with the *UAS-CycG* strains. After 24h of egg-laying, embryos were allowed to develop 24h. Then, they were heat-shocked at 37°C during 1h, allowed to develop 24h more, and heat-shocked a second time at 37°C during 1h.

The *da-Gal4*, *UAS-CycG*^*ΔP*^ third chromosome, obtained by recombination of *da-Gal4* with the original *UAS-CycG*^*ΔP*^ transgene (*RCG76*), was used to test genetic interactions between *CycG* and several *PcG* or *ETP* mutations [31]. *PcG* and *ETP* alleles used are described in Table 1.

For FA analyses, five replicate crosses were performed for each genotype, wherein 6 females carrying a Gal4 driver were mated with 5 males carrying a *CycG* transgene. Parents were transferred into a new vial every 48h (three times) then discarded. Thirty females were sampled from the total offspring of the desired genotype. For genetic interactions with *PcG* or *ETP* mutants, crosses were performed similarly except that 6 *PcG* or *ETP* mutant females were mated either with 5 *da-Gal4*, *UAS-CycG*^*ΔP*^ males, or with 5 *da-Gal4* males as control.

### Morphometrics

Right and left wings of 30 sampled females were mounted on slides, dorsal side up, in Hoyer’s medium. Slides were scanned with a Hamamatsu Nanozoomer Digital Slide scanner. Wing pictures were exported into tif format using NDP.view. All wings were oriented with the hinge to the left. Image J was used to digitize either landmarks 3 and 13 to measure wing length, or the 15 landmarks to measure more accurately wing centroid size when indicated (S2 Fig). All wings were measured twice. Analysis of wing size FA, the variance of the difference between the left and the right wing in a population, was performed as described previously [6]. The FA10 index was used to estimate FA, *i*.*e*. FA corrected for measurement error, directional asymmetry and inter-individual variation [9]. For all genotypes, the interaction individual*side was significant, indicating that FA was larger than measurement error. F-tests were performed to compare the different genotypes.

### Immunostaining of polytene chromosomes and wing imaginal discs

Immunostainings were performed as described in [31]. Primary anti-H2AK119ub antibodies (Cell Signaling D27C4) were used at a 1:40 dilution.

### RNA-seq experiments and RT-qPCR validations

Wing imaginal discs from *da-Gal4/UAS-CycG*^*ΔP*^ and *da-Gal4/+* third instar female larvae were dissected, and total RNAs were extracted as previously described except that 150 discs homogenized by pipetting were used for each extraction [58]. Three biological replicates (wing imaginal discs dissected from three independent crosses) were generated for each genotype. Library preparation and Illumina sequencing were performed at the ENS Genomic Platform (Paris, France). PolyA RNAs were purified from 1 μg of total RNA using oligo(dT). Libraries were prepared using the TruSeq Stranded mRNA kit (Illumina). Libraries were multiplexed by 6 on 2 flowcell lanes. 50 bp single read sequencing was performed on a HiSeq 1500 device (Illumina). Number of reads are shown on S17 Table. Reads were aligned with the *D*. *melanogaster* genome (dm6, r6.07) using TopHat 2 (v2.0.10) [59]. Unambiguously mapping reads were then assigned to genes and exons described by the Ensembl BDGP5 v77 assembly, by using the “summarizeOverlaps” function from the “GenomicAlignments” package (v 1.2.2) in “Union” mode [60]. Library size normalization and differential expression analysis were both performed with DESeq 2 (v 1.6.3). Genes with adjusted p-value below 0.05 were retained as differentially expressed [61]. Gene Ontology analysis was performed using DAVID [62].

For RT-qPCR validations, RNAs were extracted from wing imaginal discs and treated with Turbo DNAse (Ambion). cDNA were synthesized with SuperScript II Reverse transcriptase (Invitrogen) using random primers. RT-qPCR experiments were carried out in a CFX96 system (Bio-Rad) using SsoFast EvaGreen Supermix (Bio-Rad). Two biological replicates (cDNA from wing imaginal discs of larvae coming from independent crosses) and three technical replicates (same pool of cDNA) per biological replicate were performed for each genotype. Expression levels were quantified with the Pfaffl method [63]. The geometric mean of two reference genes, *Lamin* (*Lam*) and *rasputin* (*rin*), the expression of which did not vary when *CycG*^*ΔP*^ was expressed, was used for normalization [64]. Sequences of primer couples are listed in S16 Table.

An interactome was built using Cytoscape (v 2.8.3) and the DroID plugin (v 1.5) to introduce protein-protein and transcription factor-gene interactions [40]. The jActiveModules plugin (v 2.23) was used to find sub-networks of co-deregulated genes in the interactome by using “overlap threshold” 0.8, “score adjusted for size”, and “regional scoring” [43].

### ChIP-seq experiments and ChIP-qPCR validations

Wing imaginal discs from *+/UAS-CycG*^*ΔP*^*; da-Gal4/+* and *da-Gal4/+* third instar female larvae were used for ChIP-seq experiments. 600 wing imaginal discs were dissected (taking one disc per larva) in Schneider medium and aliquoted per 50 on ice. The 12 microtubes were treated as described in [58] with minor modifications. Discs were fixed at 22°C. 12 sonication cycles were performed (Diagenode Bioruptor sonifier; cycles of 30'' ON, 30'' OFF, high power). After centrifugation, the 12 supernatants were pooled, homogenized, and 2% were kept (Input). The remaining fragmented chromatin was redistributed into 12 tubes and each tube was adjusted to 1 mL with 140 mM NaCl, 10 mM Tris-HCl pH 8.0, 1 mM EDTA, 1% Triton X-100, 0.1% sodium deoxycholate, 0.1% BSA, Roche complete EDTA-free protease inhibitor cocktail. For immunoprecipitation, 3 μg of anti-Myc antibody (Abcam 9132) were added per tube. Two biological replicates were performed for *+/UAS-CycG*^*ΔP*^*; da-Gal4/+* and one for *da-Gal4/+*.

Library preparation and Illumina sequencing were performed at the ENS Genomic Platform (Paris, France). Libraries were prepared using NEXTflex ChIP-Seq Kit (Bioo Scientific), using 38 ng of IP or Input DNA. Libraries were multiplexed by 10 on one flowcell run. 75 bp single read sequencing was performed on a NextSeq 500 device (Illumina). Reads were filtered by the "fastq_quality_filter" command from the "fastx-Toolkit" package (http://hannonlab.cshl.edu/fastx_toolkit/), using a threshold of 90% bases with mapping quality ≥ 20. Reads are shown on S18 Table. Those that successfully passed the filtering step were aligned to the *D*. *melanogaster* genome (dm6, r6.07) using Bowtie 2 (http://bowtie-bio.sourceforge.net/bowtie2/) (v 2.1.0) with default parameters [65]. Peaks were called by MACS2 (v 2.1.0) by comparing each ChIP to its input library, with fragment size fixed at 110 bp and otherwise default parameters [66]. Peak reproducibility between the two biological replicates was then analysed with the IDR method (https://www.encodeproject.org/software/idr/) [67]. Briefly, an IDR score was assigned to each peak by the "batch-consistency-analysis" function, using the recommended parameters for MACS peaks (peak ranking based on p-value). Peaks below the 0.05 threshold were considered reproducible. The overlapping reproducible peaks from both replicates were fused using the BEDtools suite "merge" function [68], resulting in the final list of peaks kept for subsequent analysis. Cyclin G-bound genes were defined as genes from the genome annotation file (dm6, r6.07) which overlapped at least one of these Cyclin G peaks, as obtained by the BEDtools suite "intersect" function.

For ChIP-qPCR validations, ChIPs were performed similarly with the anti-Myc antibody. Rabbit IgG (Diagenode) were used as negative control (mock). qPCR experiments were carried out in a CFX96 system (Bio-Rad) using SsoFast EvaGreen Supermix (Bio-Rad). Three biological replicates–three technical replicates per biological replicate–were performed for each antibody and for the Input. Sequences of primer couples are listed in S16 Table. Data were normalized against Input chromatin.

Heatmaps and aggregation plots of Cyclin G signal over gene bodies and TSS were generated using the ngsplot package. (https://github.com/shenlab-sinai/ngsplot) [69]. Some genes with spurious signal (such as genes from the histone complex) were excluded from the analysis based on signal uniformity over the full length of the gene (cumulative derivative of Cyclin G signal over gene length = 0).

### Genomic association

Genomic loci enriched for Polycomb (Pc), Posterior Sex Comb (Psc), Polyhomeotic (Ph), RNA Polymerase II (RNAPolII) and H3K27me3 in wild type imaginal discs of third instar larvae were retrieved from GEO (GSE42106) [70–71] (H3K27me3_WholeWingDisc GSM1032567, PcRJ_AnteriorWingDisc GSM1032571, PcRJ_PosteriorWingDisc GSM1032574, Ph_WholeWingDisc GSM1032576, PolII_WholeWingDisc GSM1032577, Psc_WholeWingDisc GSM1032578. Binding sites for Pc in the whole wing disc were defined as the overlap between Pc binding sites in the anterior and posterior wing disc compartment, as obtained by the BEDtools "intersect" function. For Asx and Calypso, the bed files were a kind gift from Dr. J. Müller [34]. The mappability file for dm6 genome with 25 nt reads (the smallest size in the compared data) was generated using the Peakseq code (http://archive.gersteinlab.org/proj/PeakSeq/ Mappability_Map/Code). The overall size of the mappable genome was used as the effective genome size for the GAT software (https://github.com/AndreasHeger/gat) to assess the significance of the overlap between peaks of Cyclin G and other factors [72]. As GAT performs a two-tailed test, it displays low p-values both for significant overlap and exclusion (as between Cyclin G and H3K27me3).

Gene overlap significance assessment was made as follows: under the null hypothesis, genes that are enriched for Asx, Calypso, Pc, Psc, Ph, RNAPolII or H3K27me3 in wild type imaginal discs of third instar larvae should not exhibit any bias towards Cyclin G targets. Thus, the overlap between **n** enriched genes and **K** Cyclin G targets genes should be explained by random sampling without replacement of **n** genes within the total amount **N** of *D*. *melanogaster* genes. The amount of overlap under the null hypothesis **X** follows a hypergeometric law: *X*~*HY*(*K,N,n*). The significance of the observed overlap **k** was computed as the probability of observing **X** higher or equal to **k** under the null hypothesis: P(X ≥ k).

### Accession numbers

The data discussed in this publication have been deposited in NCBI's Gene Expression Omnibus [70] and are accessible through GEO Series accession number GSE99462 for RNA-seq, and GSE99461 for ChIP-seq.

## Supporting information

S1 Fig Western blots showing the Myc tagged transgenic proteins. Top: Membranes were stained with Ponceau red.
Bottom: The same membranes were incubated first with the anti-Myc antibody (left) or the anti-tubulin antibody (right), second with an HRP secondary antibody, then revealed with the Pierce ECL western blotting substrate.
20 μg of proteins from *da-Gal4>UAS-CycG*^*FL*^, *da-Gal4>UAS-CycG*^*ΔP*^, *da-Gal4>UAS-CycG*^*ΔEΔP*^, *da-Gal4>UAS-CycG*^*ΔE*^, or *yw* third instar larvae were loaded per track.
(TIF)

S2 Fig Acquisition of morphometric data. Red dots show the 15 landmarks digitized on the wings. The coordinates of these landmarks were obtained from the left and right wings of 30 females randomly sampled from a population. FA was expressed using the FA10 index, *i*.*e*. the variance of the difference between the left and the right wings in the population, corrected for the measurement error, directional asymmetry and inter-individual variances.
(TIF)

S1 Table Wing length fluctuating asymmetry of flies expressing *CycG*^*ΔP*^ with different Gal4 drivers. Wing length fluctuating asymmetry was estimated with the FA10 index using landmarks 3 and 13 as described previously [6]. See source data in S2 Table. Standard F-tests were used to compare FA values between genotypes. Df: degrees of freedom. *CycG*^*ΔP*^: cDNA encoding the protein deleted of the PEST domain. n: number of females measured.
(XLS)

S2 Table Source data for S1 Table. Length (from landmarks 3 to 13) of left (side 1) and right (side 2) wings. Each wings were measured twice (sessions 1 and 2).
(XLS)

S3 Table Wing centroid size fluctuating asymmetry of flies expressing different versions of Cyclin G. Wing centroid size fluctuating asymmetry was estimated with the FA10 index using the 15 landmarks as described previously [6]. See source data in S4 Table. Standard F-tests were used to compare FA values between genotypes. Df: degrees of freedom. n: total number of females analysed. *CycG*^*FL*^: cDNA encoding the full-length protein; *CycG*^*ΔE*^: cDNA encoding a protein deleted of the ETP-interacting domain; *CycG*^*ΔP*^: cDNA encoding a protein deleted of the PEST domain; *CycG*^*ΔEΔP*^: cDNA encoding a protein deleted of both domains.
(XLS)

S4 Table Source data for S3 Table. Coordinates of the 15 landmarks of left (side 1) and right (side 2) wings. Each wings were measured twice (sessions 1 and 2).
(XLS)

S5 Table Wing centroid size fluctuating asymmetry of flies expressing *CycG*^*ΔP*^ combined with different *PcG* or *ETP* mutant alleles. Wing centroid size fluctuating asymmetry was estimated with the FA10 index using the 15 landmarks as described previously [6]. See source data in S6 Table. Standard F-tests were used to compare FA values between genotypes. Df: degrees of freedom.
(XLS)

S6 Table Source data for S5 Table. Coordinates of the 15 landmarks of left (side 1) and right (side 2) wings. Each wing was measured twice (sessions 1 and 2).
(XLS)

S7 Table List of the 530 genes deregulated in *da-Gal4/+*, *UAS- CycG*^*ΔP*^ wing imaginal discs as compared to *da-Gal4/+* wing imaginal discs. (XLS)

S8 Table Measure of endogenous *CycG* expression by RT-qPCR. AE: amplification efficiency of the primer couples. Expression of *CycG* was normalized on the geometric mean of *Lam* and *rin* (chosen as reference genes as their expression was not modified by *CycG*^*ΔP*^ expression). Two biological replicates (called 1 and 2) and three technical replicates were performed per experiment. t-tests were performed to compare expression of *CycG* in *da-Gal4*, *UAS-CycG*^*ΔP*^*/+* and *da-Gal4/+* wing imaginal discs.
(XLS)

S9 Table Ontology of genes deregulated in *UAS-CycG*^*ΔP*^, *da-Gal4/+* wing imaginal discs. Gene ontology analyses were performed with DAVID (https://david.ncifcrf.gov/home.jsp).
(XLS)

S10 Table Validation of RNA-seq experiments by RT-qPCR. AE: amplification efficiency of the primer couples. Expression of *RPL15*, *RPL7* and *Rack1* were normalized on the geometric mean of *Lam* and *rin* (chosen as reference genes as their expression was not modified by *CycG*^*ΔP*^ expression). Two biological replicates (called 1 and 2) and three rechnical replicates were performed per experiment. t-tests were performed to compare expression of these genes in *da-Gal4*, *UAS-CycG*^*ΔP*^*/+ and da-Gal4/+* wing imaginal discs.
(XLS)

S11 Table List of the 889 genes which Transcriptional Start Site is bound by Cyclin G in wing imaginal discs. (XLS)

S12 Table Repartition of feature types among decile-ranked peaks. (XLS)

S13 Table Validation of ChIP-seq experiments by RT-qPCR. AE: amplification efficiency of the primer couples. Cq of the Input were adjusted taking dilution into account. Results were normalized in comparison to the Input. Three biological replicates (named 1, 2 and 3) and three technical replicates per biological replicate were performed.
(XLS)

S14 Table List of the 62 genes deregulated in *da-Gal4*, *UAS-CycG*^*ΔP*^*/+* wing imaginal discs and bound by Cyclin G at the TSS. (XLS)

S15 Table Comparison of fragments bound by Cyclin G with fragments bound by Asx, Calypso, Pc, Ph, Psc, RNAPolII, or enriched in H3K27me3 in 3rd larval instar wing imaginal discs. (XLS)

S16 Table Primers used in this study. Coordinates on the *Drosophila* genome (dm6, r6.13). F: forward primer, R: reverse primer.
(XLS)

S17 Table RNA-seq of wing imaginal discs. (XLS)

S18 Table ChIP-seq of wing imaginal discs. (XLS)

S1 File WID.zip file. Wing imaginal disc (WID) network composed of 9,966 nodes connected *via* 56,133 edges (WID.xmml).
(ZIP)

S2 File CycG_subnetwork.zip file. Sub-network of 222 nodes and 1069 edges centred on Cyclin G (CycG_subnetwork.xmml).
(ZIP)
