# Supplementary figures and images for: Cyclin G and the Polycomb Repressive complexes PRC1 and PR-DUB cooperate for developmental stability
Source: PLoS Genet. 2018 Jul 11;14(7):e1007498. doi: 10.1371/journal.pgen.1007498 (PMC6065198; doi:10.1371/journal.pgen.1007498)

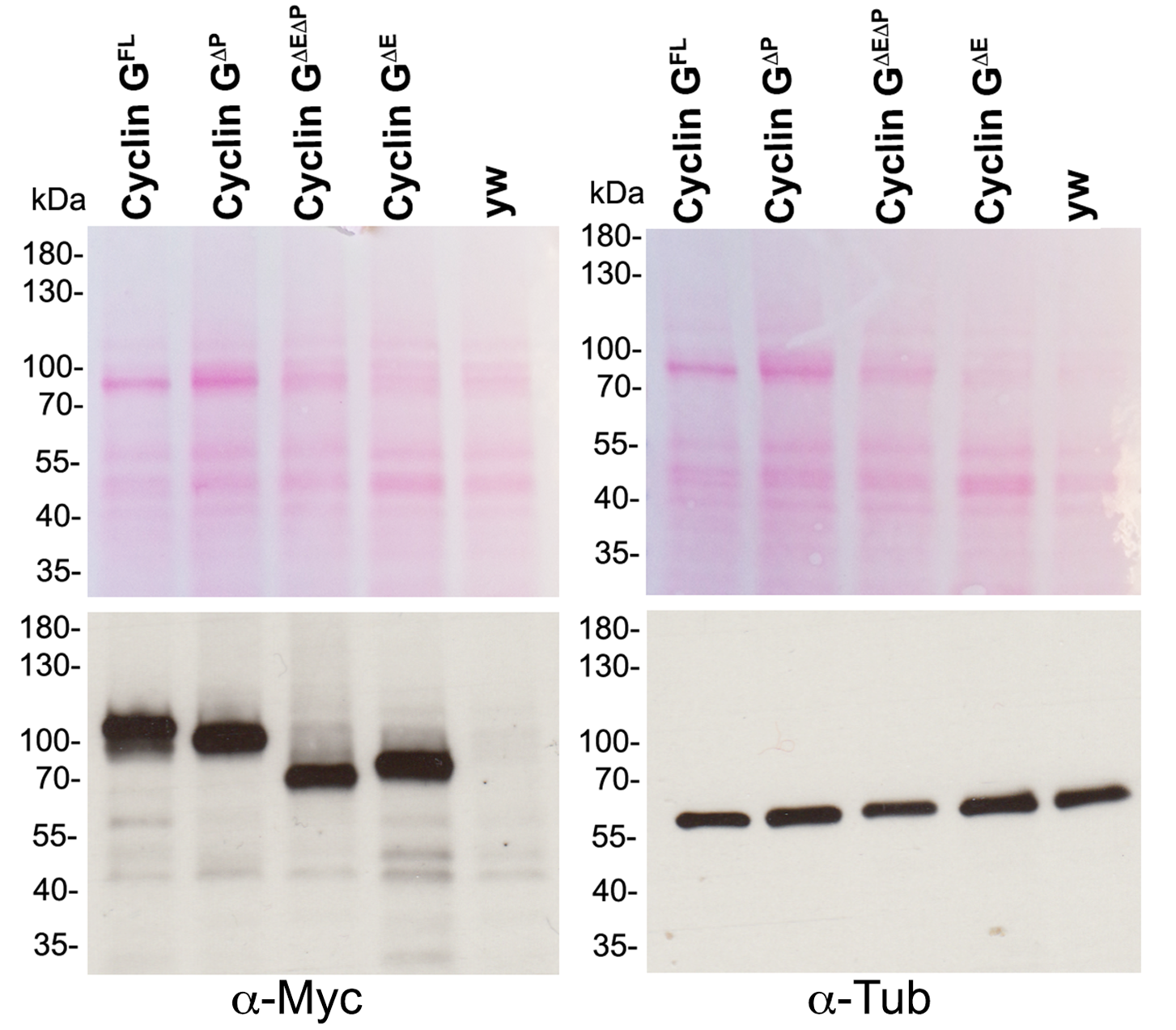

Supplement: S1 Fig — Top: Membranes were stained with Ponceau red. Bottom: The same membranes were incubated first with the anti-Myc antibody (left) or the anti-tubulin antibody (right), second with an HRP secondary antibody, then revealed with the Pierce ECL western blotting substrate. 20 μg of proteins from da-Gal4>UAS-CycGFL, da-Gal4>UAS-CycGΔP, da-Gal4>UAS-CycGΔEΔP, da-Gal4>UAS-CycGΔE, or yw third instar larvae were loaded per track. (TIF) [file pgen.1007498.s001.tif]

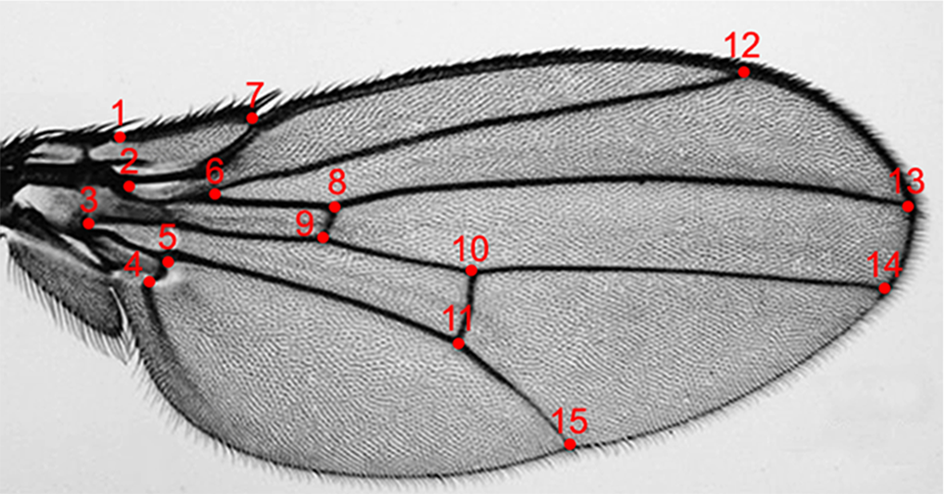

Supplement: S2 Fig — Red dots show the 15 landmarks digitized on the wings. The coordinates of these landmarks were obtained from the left and right wings of 30 females randomly sampled from a population. FA was expressed using the FA10 index, i.e. the variance of the difference between the left and the right wings in the population, corrected for the measurement error, directional asymmetry and inter-individual variances. (TIF) [file pgen.1007498.s002.tif]
